# A novel octavalent combined Erysipelas, Parvo and Leptospira vaccine provides (cross) protection against infection following challenge of pigs with 9 different *Leptospira interrogans* serovars

**DOI:** 10.1186/s40813-015-0011-0

**Published:** 2015-11-17

**Authors:** A. Jacobs, F. Harks, M. Hoeijmakers, R. Segers

**Affiliations:** MSD Animal Health, Wim de Körverstraat 35, PO Box 31, 5830 AA Boxmeer, The Netherlands

**Keywords:** Swine, Leptospirosis, Erysipelas, Parvovirus, Vaccine, Cross protection

## Abstract

**Background:**

In the present study the efficacy and cross protection of a novel ready-to-use combination vaccine, Porcilis® Ery + Parvo + Lepto, against swine leptospirosis, was investigated. The octavalent vaccine contains inactivated antigens of *Erysipelothrix rhusiopathiae*, Parvovirus and 6 *Leptospira interrogans sensu lato* strains of serogroups Canicola, Icterohaemorrhagiae, Grippotyphosa, Australis (Bratislava), Pomona and Tarassovi. In this study ninety pigs were vaccinated twice with Porcilis® Ery + Parvo + Lepto at six and ten weeks of age and ninety age and source-matched animals served as unvaccinated control. Two weeks after booster vaccination, groups of vaccinated and control pigs (ten pigs per group) were challenged with fresh virulent cultures of either of the nine different challenge strains. Compared to the vaccine strains the challenge strains were heterologous strains of the same serovar or of a different serovar within the same serogroup. The challenge strains tested were of serovar Canicola, Icterohaemorrhagiae, Copenhageni (serogroup Icterohaemorrhagiae), Grippotyphosa, Bananal/Liangguan (serogroup Grippotyphosa), Pomona, Tarassovi and Vughia (serogroup Tarassovi).

**Results:**

After the different challenges most control animals became leptospiraemic for 2–7 days. The vaccinated pigs remained blood culture negative except for two animals after serovar Icterohaemorrhagiae and two animals after serovar Tarassovi challenge which became leptospiraemic for only 1 day. The incidence of Leptospiraemia (as determined by blood culture) was significantly less in vaccinates compared to the controls after all challenges. The vaccine also prevented renal infection and urinary shedding after serovar Canicola challenge. The other serovars did not induce detectable renal infection or urinary shedding.

**Conclusion:**

The present study demonstrates that the new combination vaccine Porcilis® Ery + Parvo + Lepto induces significant (cross) protection against nine different serovars within the serogroups Canicola, Icterohaemorrhagiae, Grippotyphosa, Australis (Bratislava), Pomona and Tarassovi.

## Background

Leptospirosis, caused by *Leptospira interrogans sensu lato*, is a cause of reproductive failure in pigs worldwide, which manifests itself as abortions or the birth at term of a variable number of mummified, autolysed, stillborn and/or weak piglets [1–4]. Leptospirosis in pigs, as in other animals and humans, is difficult to diagnose and its incidence is most probably underestimated. Culture, serology and PCR often are negative even when active infection is present. In addition, in sows, the clinical signs of *Leptospira* infection are few, vague and non-specific or absent.

Reports on swine relevant serovars are scars and the few reports that are available indicate that serogroups Pomona, Tarassovi, Australis (Bratislava), Grippotyphosa, Icterohaemorrhagiae and Canicola are most commonly isolated from pigs with reproductive problems [3–7].

Recently, we reported on the development of a new ready-to-use combination product (Porcilis® Ery + Parvo + Lepto), containing inactivated *Erysipelothrix rhusiopathiae*, Parvovirus, and *Leptospira interrogans sensu lato* serogroups Canicola, Icterohaemorrhagiae, Australis (Bratislava), Grippotyphosa, Pomona and Tarassovi [8]. In that study it was demonstrated that the vaccine can be safely used in gilts and sows and induces significant protection, for the duration of at least one year, against serovar Pomona induced clinical signs, leptospiraemia and foetal death. In addition, protection against Pomona associated reproductive failure was confirmed under field conditions where a significant reduction in abortion rate was observed.

It is generally assumed that *Leptospira* vaccines induce protection within serogroups, i.e. that one serovar induces cross-protection against other serovars within the same serogroup but evidence, especially in pigs, is limited [9, 10]. The objective of the present study was to evaluate efficacy of the new combination vaccine Porcilis® Ery + Parvo + Lepto against nine different homologous as well as heterologous *Leptospira* serovar challenge strains.

## Results

### Serology

On the day of challenge the vaccinated pigs had various levels of serogroup specific microscopic agglutination (MAT) titres whereas the controls remained seronegative (Table 1). Highest responses were found for serogroups Canicola and Australis (Bratislava) whereas the serogroups Icterohaemorrhagiae (serovar Copenhageni) and Tarassovi (serovar Gatuni) vaccine strains showed only poor antibody responses after vaccination. Four weeks after challenge, most pigs showed high MAT titres except for the serovar Grippotyphosa and serovar Tarassovi challenge controls which showed a poor response.

**Table 1:** Average MAT titres after vaccination and challenge. Pigs were vaccinated twice (4-week interval) and challenged with different *Leptospira* serovar (sv) and serogroup (sg) challenge strains, 6 weeks after first vaccination. MAT titres homologous to challenge strain

			avg MAT titres ± SD (log_2_), weeks after first vaccination			
n	group	Challenge with	0	4	6	10
9	vaccine	sv Canicola	<2	<2	9.6 ± 2.0	9.6 ± 1.3
10	control	sg Canicola	<2	<2	<2	10.3 ± 1.1
10	vaccine	sv Copenhageni	<2	<2	3.8 ± 1.2	9.6 ± 1.0
10	control	sg Icterohaemorrhagiae	<2	<2	<2	10.3 ± 0.7
10	vaccine	sv Icterohaemorrhagiae	<2	<2	<2	9.1 ± 3.3
10	control	sg Icterohaemorrhagiae	<2	<2	<2	8.7 ± 1.4
10	vaccine	sv Bananal / Liangguan	<2	<2	6.9 ±1.4	9.3 ± 0.8
9	control	sg Grippotyphosa	<2	<2	<2	9.1 ± 0.8
8	vaccine	sv Grippotyphosa	<2	<2	4.6 ± 2.4	7.6 ± 1.3
10	control	sg Grippotyphosa	<2	<2	<2	2.0 ± 1.8
10	vaccine	sv Bratislava	<2	6.9 ± 1.2	10.4 ± 0.8	10.5 ± 1.1
9	control	sg Australis	<2	<2	<2	9.9 ± 1.2
9	vaccine	sv Pomona	<2	<2	5.0 ± 4.0	6.7 ± 3.2
10	control	sg Pomona	<2	<2	<2	9.6 ± 0.8
10	vaccine	sv Vughia	<2	<2	<2	8.5 ± 0.8
10	control	sg Tarassovi	<2	<2	<2	9.5 ± 1.0
10	vaccine	sv Tarassovi	<2	<2	2.0 ± 3.2	8.0 ± 1.3
10	control	sg Tarassovi	<2	<2	<2	4.3 ± 1.3

### Clinical signs and rectal temperature

**Fig. 1:**
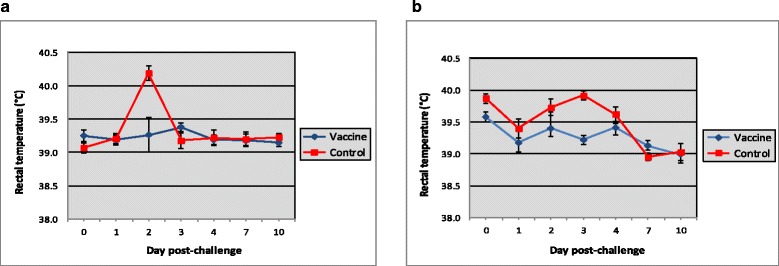
Time-course of average rectal temperature (± sem) after challenge. Groups of ten pigs were vaccinated twice, 4 weeks apart, with Porcilis^®^ Ery + Parvo + Lepto (vaccinates) or were left unvaccinated (controls). Two weeks after booster vaccination, pigs were challenged with *Leptospira* serovar Icterohaemorrhagiae (**a**) or serovar Canicola (**b**)

An increase in rectal temperature was found on post-challenge day 2 or 3 in control animals after serovar Icterohaemorrhagiae and serovar Canicola challenge, respectively (Fig. 1). These temperature effects (AUC) were significantly different between vaccinates and controls; *p* = 0.012 and *p* = 0.010 after serovar Icterohaemorrhagiae and serovar Canicola challenge, respectively. After the other serovar challenges no effects on rectal temperature were observed. Further no challenge related clinical signs were observed (not shown).

### Re-isolation of challenge organisms from blood

After the different challenges most control animals became leptospiraemic for 2–7 days (Table 2). The vaccinated pigs remained blood culture negative except for two animals after serovar Icterohaemorrhagiae and 2 pigs after serovar Tarassovi challenge which became leptospiraemic for only 1 day. The number of pigs infected was significantly less in vaccinates compared to controls (Fisher’s exact) after all challenges except after serovar Tarassovi challenge where 2/10 vaccinates were infected compared to 6/10 controls (*p* = 0.1698). However, the number of positive blood isolations was significantly reduced (GEE) after challenge with this serovar (*p* = 0.0285).

**Table 2:** Reisolation of Leptospira from blood. Pigs were vaccinated twice (4-week interval) and challenged with different Leptospira serovar (sv) and serogroup (sg) challenge strains, 6 weeks after first vaccination. A pig was considered infected if at least once a positive blood isolation was found. n.a. = not applicable

			reisolation of Leptospira from blood on post-challenge day							# pigs infected	# blood isolations
n	group	challenge with	0	1	2	3	4	7	10		
9	vaccine	sv Canicola	0	0	0	0	0	0	0	0**	n.a.
10	control	sg Canicola	0	10	10	10	10	1	0	10	n.a.
10	vaccine	sv Copenhageni	0	0	0	0	0	0	0	0**	n.a.
10	control	sg Icterohaemorrhagiae	0	10	10	8	4	1	0	10	n.a.
10	vaccine	sv Icterohaemorrhagiae	0	2	0	0	0	0	0	2**	2**
10	control	sg Icterohaemorrhagiae	0	9	6	2	0	0	0	9	17
10	vaccine	sv Bananal / Liangguan	0	0	0	0	0	0	0	0**	n.a.
9	control	sg Grippotyphosa	0	6	8	6	2	0	0	8	n.a.
8	vaccine	sv Grippotyphosa	0	0	0	0	0	0	0	0**	n.a.
10	control	sg Grippotyphosa	0	6	1	0	0	0	0	6	n.a.
10	vaccine	sv Bratislava	0	0	0	0	0	0	0	0**	n.a.
9	control	sg Austrais	0	6	4	0	0	0	0	6	n.a.
9	vaccine	sv Pomona	0	0	0	0	0	0	0	0**	n.a.
10	control	sg Pomona	0	10	10	10	9	2	0	10	n.a.
10	vaccine	sv Vughia	0	0	0	0	0	0	0	0**	n.a.
10	control	sg Tarassovi	0	10	9	1	1	0	0	10	n.a.
10	vaccine	sv Tarassovi	0	2	0	0	0	0	0	2	2*
10	control	sg Tarassovi	0	6	2	1	1	0	0	6	10

* *p<*0.05, ** *p<*0.01

### Re-isolation of challenge organisms from urine and kidney

With the exception of serovar Canicola and serovar Icterohaemorrhagiae all urine cultures remained culture negative. After Canicola challenge all ten control animals showed leptospiruria on one or more days (Table 3). Also six kidney cultures became positive in the control group. The urine and kidney cultures of the vaccine group remained negative. Both the number of pigs shedding and the number of positive kidney cultures were significantly different between vaccinates and controls.

**Table 3:** Reisolation of Leptospira from urine and kidney. Pigs were vaccinated twice (4-week interval) and challenged with different *Leptospira* serovar (sv) and serogroup (sg) challenge strains, 6 weeks after first vaccination. Urine was sampled regularly and kidney samples were collected during necropsy 4w after challenge. A pig was considered shedding if at least once a positive urine isolation was found

			reisolation of Leptospira from urine on post-challenge day						# pigs shedding	# Kidney positive
n	group	challenge with	0	14	17	21	24	28		
9	vaccine	sv Canicola	0	0	0	0	0	0	0**	0**
10	control	sg Canicola	0	9	9	9	7	6	10	6
10	vaccine	sv Copenhageni	0	0	0	0	0	0	0	0
10	control	sg Icterohaemorrhagiae	0	0	0	0	1	1	1	0

** *p<*0.01

After Icterohaemorrhagiae challenge only one control animal became urine culture positive and all kidney cultures remained negative.

## Discussion

In this study, all different serovar challenge strains induced leptospiraemia in the controls. An increased rectal temperature was only measured after serovar Canicola and serovar Icterohaemorrhagiae challenge and significant leptospiruria was only observed after serovar Canicola challenge. The new octavalent combination vaccine protected against those phenomena and induced cross-protection between different serovars within serogroups. In addition, we demonstrated cross-protection within serogroups across species level as the serogroup Tarassovi vaccine strain and the two Tarassovi challenge strains represent three different species; *Leptospira santarosai, Leptospira weilii and Leptospira borgpetersenii*, respectively.

After vaccination remarkable differences in serological responses were found. The vaccine induced high serogroup specific MAT titres against Canicola and Australis whereas MAT titres to serogroups Tarassovi and Icterohaemorrhagiae appeared either absent or very low. Even in the absence of detectable antibodies the vaccine protected against challenge with two different serovars of serogroup Tarassovi and two different serovars of serogroup Icterohaemorrhagiae, indicating that detectable MAT titres are not required for protection. This is consistent with results of Whyte et al. [11] who also reported protection against Pomona in the absence of significant Pomona MAT titres. It is possible that very low concentrations (below the limit of quantification of the assay) of serum agglutinating antibodies may protect against infection. This is in line with several earlier studies [12–14] which showed that sera from vaccinated animals or humans with low or undetectable concentrations of agglutinating antibodies afforded protection in the passive hamster protection test.

Striking differences in serological responses were also found after infection of control animals. Most serovars induced high MAT titres after challenge. However, serovar Grippotyphosa and serovar Tarassovi hardly induced MAT titres whereas other serovars within the Grippotyphosa and Tarassovi serogroups (Bananal/Liangguang and Vughia, respectively) induced high titres. This difference may be explained by the observation that the first two strains appeared less virulent and had a shorter duration of leptospiraemia. This result underlines the limited value of serology in diagnosing *Leptospira* related problems in pigs as pigs can be infected without showing a serological response.

Except for serovar Canicola renal infection or leptospiruria was not observed in this study. This is in contrast to the results of Hodges et al. [2] and Whyte et al. [11] who found urinary shedding after serovar Pomona challenge and Ellis et al. [15] who found renal infection after serovar Bratislava challenge. This difference may be due to the differences in age or susceptibility of the pigs, differences in the challenge strains or a different detection limit between the diagnostic methods.

In this study the vaccine completely prevented renal leptospirosis as well as urinary shedding after serovar Canicola challenge. Hodges [16] also observed prevention of leptospiruria after four repeated vaccinations and a natural serovar Pomona challenge whereas in the studies described by Hodges et al. [2] and Whyte et al. [11] neither vaccine did prevent urinary shedding after serovar Pomona challenge. Prevention of Leptospiruria is an important feature of a successful vaccine as urinary shedding is the main source of transmission to naïve animals and men.

## Conclusion

The present study demonstrates that the new combination vaccine Porcilis® Ery + Parvo + Lepto induces significant (cross) protection against nine different serovars within the serogroups Canicola, Icterohaemorrhagiae, Grippotyphosa, Australis (Bratislava), Pomona and Tarassovi.

## Methods

### Vaccine

A vaccine containing inactivated *Erysipelothrix rhusiopathiae,* Parvo virus, *Leptospira interrogans (sensu lato)* serogroups Canicola, Icterohaemorrhagiae, Australis (Bratislava), Grippotyphosa, Pomona and Tarassovi and Diluvac Forte® adjuvant (Porcilis® Ery + Parvo + Lepto, MSD Animal Health).

### Leptospira strains

The vaccine strains and challenge strains evaluated in this study are shown in Table 4. All strains were obtained at an unknown passage level from Dr. C. Bolin, (USDA, NADC,USA), Dr R. Hartskeel (KIT, Amsterdam, The Netherlands), Public Health Laboratory Service, UK or from Queensland health Science Services, Australia.

**Table 4:** Leptospira strains used for vaccine / challenge

Species	Serogroup	Serovar	Strain	Originally isolated from
*Leptospira interrogans*	Canicola	Portland-vere^a^	Ca-12-000	human blood, 1964, Jamaica
*Leptospira interrogans*		Canicola^b^	Moulton	pig urine, 2004, Netherlands
*Leptospira interrogans *	Icterohaemorrhagiae	Copenhageni^a^	Ic-02-001	rat kidney, 1978, USA
*Leptospira interrogans*		Copenhageni^b^	CF1	dog, 1969, Puerto Rico
Leptospira interrogans		Icterohaemorrhagiae^b^	Verdun	human, 1917, France
*Leptospira kirschneri*	Grippotyphosa	Dadas^a^	Gr-01-005	kidney aborted piglet, 1983, USA
*Leptospira kirschneri*		Bananal/Lianguang^b^	11808	shrew, 1972, USA
*Leptospira kirschneri*		Grippotyphosa^b^	142	horse eye, 1997 Germany
*Leptospira interrogans*	Australis	Bratislava^a^	As-05-073	pig placenta, 1989, USA
*Leptospira interrogans*		Bratislava^b^	X35IM-001	pig, 1990, USA
*Leptospira interrogans*	Pomona	Pomona^a^	Po-01-000	human blood, 1937, Australia
*Leptospira interrogans*		Pomona^b^	02-0162	not known
*Leptospira santarosai*	Tarassovi	Gatuni^a^	X345	human blood, 1938, Russia
*Leptospira weilii*		Vughia^b^	L100	pig kidney, 2001, China
*Leptospira borgpetersenii*		Tarassovi^b^	Perepelitsin	human blood, 1941, Russia

^a^vaccine strain

^b^challenge strain

After arrival the strains had 2–3 EMJH medium passages after which they were stored in liquid nitrogen. The vaccine strains had 5–7 further medium passages before they were used in the vaccine. The challenge strains were either used after two additional medium passages (serovars Grippotyphosa, Bratislava, Vughia and Tarassovi) or after hamster passage followed by two additional medium passages (serovars Canicola, Copenhageni, Icterohaemorrhagiae, Bananal/Lianguang, Pomona).

### Study design

Nine different vaccination-challenge trials were performed. In total one hundred and eighty 6-week-old pigs, with undetectable levels of serum antibodies against *Leptospira* serogroups Canicola, Icterohaemorrhagiae, Australis (Bratislava), Grippotyphosa, Pomona and Tarassovi, were selected. Ninety pigs were vaccinated twice with Porcilis® Ery + Parvo + Lepto with a 4-week interval between the vaccinations. The other ninety pigs were left unvaccinated and served as challenge controls. Two weeks after booster vaccination, groups of vaccinated and control pigs (ten pigs per group) were challenged with fresh cultures of one of the nine different virulent *Leptospira* challenge strains (10^9^ bacteria/ml). Challenge was done by intra-peritoneal (IP) injection of 20 ml and conjunctival instillation (both eyes) of 0.25 ml culture per eye. The conjunctival challenge was repeated one day later. The challenge strains used were heterologous isolates of the same serovar as well as heterologous serovar strains within the same serogroup compared to the vaccine strains (Table 4).

Blood samples were taken for serology, using the microscopic agglutination test (MAT), after vaccination and challenge. Blood and urine samples were taken before and after challenge to isolate the challenge strain. The pigs were observed regularly for clinical signs, including lethargy and anorexia for up to 4 weeks. Rectal temperature was measured just before challenge and on days 1, 2, 3, 4, 7 and 10 after challenge.

The pigs were euthanized 4 weeks after challenge. Necropsy was performed and the internal organs were inspected for abnormalities. Kidney samples were taken for re-isolation of challenge organisms.

Before challenge six animals were culled because of aspecific causes such as gastric torsion, pericarditis and locomotory problems, implying that a few challenge groups consisted of less than ten animals.

### Serology

Blood samples were collected from each pig on the day of first vaccination, day of booster vaccination, day of challenge and 4 weeks after challenge into Serum Sep. Clot Activator tubes (without anticoagulant for preparation of serum). The serum samples were stored frozen until analysis. Serogroup specific agglutination titres were determined in the microscopic agglutination (MAT) test as described previously [17]. Titres <2 log_2_ are regarded as negative. For calculation purposes <2 was replaced by 1.

### Isolation of challenge organisms from blood or urine

To isolate the challenge strain, blood samples were taken into heparinized tubes, just before challenge and on days 1, 2, 3, 4, 7 and 10 post-challenge. Urine was sampled just before challenge and on days 14, 17, 21, 24 and 28 post-challenge for re-isolation of challenge strain.

Blood (heparinized) or urine samples (0.5 ml) were added to 10 ml of liquid EMJH medium containing 200 μg/ml 5-fluorouracil and 1 % (v/v) rabbit serum negative for agglutinating antibodies against the 6 different serovars included in the vaccine. The cultures were incubated at 28–30 °C, and observed fortnightly using dark-field microscopy for a total of at least 8 weeks before negative cultures were discarded. The identity of the isolates was confirmed in the MAT test using specific anti-sera.

### Isolation of challenge organisms from kidney

At necropsy a 1–2 g sample was taken from the renal cortex of one kidney of each pig. The kidney samples were put into liquid EMJH medium (containing 5-fluorouracil and negative rabbit serum) and homogenised with an Ultraturrax homogeniser. A 100-fold dilution of kidney homogenate was cultured in EMJH and examined as described above.

### Statistical analysis

The level of significance α was set at 0.05 and all tests were two sided. Statistical analyses were carried out using the statistical programme SAS V9.1 or higher (SAS Institute Inc. Cary NC, USA).

#### Rectal temperature

The time course of the temperature responses following challenge was visually inspected. Only in case the controls had a response after challenge, the effect of vaccination on rectal temperature was evaluated. The Area under the Curve (AUC) of the rectal temperature over time was calculated by the linear trapezoidal rule and using the pre-challenge data as baseline [18, 19]. The AUC was analysed by Analysis of Variance (ANOVA).

#### Bacterial isolation from blood

The proportion of infected pigs, defined as a pig having at least one positive blood re-isolation after challenge, was analyzed by Fisher’s exact test and the relative percentage protection was derived from the formula (1 − % infected Vaccine / % infected Control) × 100 %. In studies where in the vaccinated pigs not all post-challenge blood samples remained negative, the incidence of a positive blood sample was analyzed by Generalized Estimating Equations (GEE), accounting for the repeated measurements structure of the data [20] and using a log binomial regression model to estimate the relative risk and derived relative percentage protection [20, 21]. The p-value was based on the empirical standard error.

#### Bacterial isolation from kidney and urine

Bacterial re-isolation data from urine and kidney, categorized as positive or negative were evaluated by Fisher’s exact test [19].

## Abbreviations

AUC: area under the curve
Ery: *Erysipelothrix rhusiopathiae*
GEE: Generalized Estimating Equations
MAT: microscopic agglutination test
SD: standard deviation
SEM: standard error of the mean

## References

[1] Wrathall AE (1975). Reproductive disorders in Pigs, rev. series no. 11.

[2] Hodges RT, Stocker RP, Buddle JR (1976). *Leptospira interrogans* serotype Pomona infection and Leptospiruria in vaccinated pigs. N. Z. Vet. J.

[3] Ellis WA, Straw BE, Zimmerman JJ, D'Allaire S, Taylor DJ (2006). Leptospirosis, chapter 41, Diseases of Swine.

[4] Gresham A (2003). Infectious reproductive disease in pigs. In Practice.

[5] The Merck Veterinary Manual. Leptospirosis in swine by T. J. Divers, April 2015, http://www.merckvetmanual.com/mvm/generalized_conditions/leptospirosis/leptospirosis_in_swine.html

[6] The pig site. Leptospirosis. http://www.thepigsite.com/pighealth/article/451/leptospirosis/

[7] Bolin CA (1994). Diagnosis of Leptospirosis in swine. SHAP: Diagnostic Notes.

[8] Jacobs AAC, Harks H, Hoeijmakers M, Collell M, Segers RPAM (2015). Safety and efficacy of a new octavalent combined Erysipelas, Parvo and *Leptospira* vaccine in gilts against *Leptospira interrogans* serovar Pomona associated disease and foetal death. Vaccine..

[9] Klaasen HLBM, van der Veen M, Molkenboer MJCH, Sutton D (2013). A novel tetravalent *Leptospira* bacterin protects against infection and shedding following challenge in dogs. Vet. Rec..

[10] Dib CC, Gonçales AP, de Morais ZM, de Souza GO, Miranglia F, Abreu PAE, Vasconcellos SA (2014). Cross-protection between experimental anti-leptospirosis bacterins. Brazilian J. Microbiol..

[11] Whyte P, Ratcliff R, Cargill C, Dobson K (1982). Protection of pregnant swine by vaccination against *Leptospira* infection. Aus. Vet. J..

[12] Morsi HM, Shibley GP, Strother HL (1973). Antibody response of swine to Leptospira Canicola and Leptospira Icterohaemorrhagiae. Am. J. vet. Res..

[13] Bet RF, Johnson RC (1978). Humoral immune response of dogs vaccinated with leptospiral pentavalent outer envelope and whole culture vaccines. Am. J. Vet. Res..

[14] Rodriguez-Gonzales I, Fillonneau C, Blanchet B, Suard I, Catilina P, André-Fontaine G (2004). Efficacy of the Spirolept vaccine against human leptospirosis estimated by passive sera-protection of laboratory rodents. Méd. Mal. Infect..

[15] Ellis WA, Montgomery JM, McParland PJ (1989). An experimental study with a Leptospira interrogans serovar. Bratislava vaccine..

[16] Hodges RT. *Leptospira interrogans* serotype Pomona infection in pigs: prevention of Leptospiruria by immunisation before exposure to a natural infection. N Z Vet J. 1977;25:33–35. 10.1080/00480169.1977.34346 275680

[17] Faine S (1994). *Leptospira* and Leptospirosis.

[18] Verbeke G, Molenberghs G (2000). Linear mixed models for longitudinal data.

[19] Agresti A (2002). Categorical Data Analysis.

[20] SAS support centre. http://support.sas.com/kb/23/003.html. Accessed 24 March 2015

[21] Spiegelman D, Easy HE, SAS (2005). Calculations for Risk or Prevalence Ratios and Differences. Am. J. Epidemiol.

