# Erratum to: A novel octavalent combined Erysipelas, Parvo and Leptospira vaccine provides (cross) protection against infection following challenge of pigs with 9 different *Leptospira interrogans* serovars

**DOI:** 10.1186/s40813-015-0014-x

**Published:** 2015-12-22

**Authors:** A. Jacobs, F. Harks, M. Hoeijmakers, R. Segers

**Affiliations:** MSD Animal Health, Wim de Körverstraat 35, Boxmeer, 5830 AA The Netherlands

## Erratum

The tables and their references published in the original version of this article [1] were regretfully incorrectly typeset. The correct tables and table citations have been updated in the original article. The correct tables have also been published in this Erratum for quick reference.

**Table 1:** Average MAT titres after vaccination and challenge. Pigs were vaccinated twice (4-week interval) and challenged with different *Leptospira* serovar (sv) and serogroup (sg) challenge strains, 6 weeks after first vaccination. MAT titres homologous to challenge strain

			avg MAT titres ± SD (log_2_), weeks after first vaccination			
n	Group	Challenge with	0	4	6	10
9	vaccine	sv Canicola	<2	<2	9.6 ± 2.0	9.6 ± 1.3
10	control	sg Canicola	<2	<2	<2	10.3 ± 1.1
10	vaccine	sv Copenhageni	<2	<2	3.8 ± 1.2	9.6 ± 1.0
10	control	sg lcterohaemorrhagiae	<2	<2	<2	10.3 ± 0.7
10	vaccine	sv lcterohaemorrhagiae	<2	<2	<2	91 ± 3.3
10	control	sg lcterohaemorrhagiae	<2	<2	<2	8.7 ± 1.4
10	vaccine	sv Bananal / Liangguan	<2	<2	6.9 ± 1.4	9.3 ± 0.8
9	control	sg Grippotyphosa	<2	<2	<2	91 ± 0.8
8	vaccine	sv Grippotyphosa	<2	<2	4.6 ± 2.4	7.6 ± 1.3
10	control	sg Grippotyphosa	<2	<2	<2	2.0 ± 1.8
10	vaccine	sv Bratislava	<2	6.9 ± 1.2	10.4 ± 0.8	10.5 ± 1.1
9	control	sg Australis	<2	<2	<2	9.9 ± 1.2
9	vaccine	sv Pomona	<2	<2	5.0 ± 4.0	6.7 ± 3.2
10	control	sg Pomona	<2	<2	<2	9.6 ± 0.8
10	vaccine	sv Vughia	<2	<2	<2	8.5 ± 0.8
10	control	sg Tarassovi	<2	<2	<2	9.5 ± 1.0
10	vaccine	sv Tarassovi	<2	<2	2.0 ± 3.2	8.0 ± 1.3
10	control	sg Tarassovi	<2	<2	<2	4.3 ± 1.3

**Table 2:** Reisolation of Leptospira from blood. Pigs were vaccinated twice (4-week interval) and challenged with different *Leptospira* serovar (sv) and serogroup (sg) challenge strains, 6 weeks after first vaccination. A pig was considered infected if at least once a positive blood isolation was found. n.a. = not applicable

			Reisolation of Leptospira from blood on post-challenge day							# pigs infected	# blood isolations
n	Group	Challenge with	0	1	2	3	4	7	10		
9	vaccine	sv Canicola	0	0	0	0	0	0	0	0^**^	n. a.
10	control	sg Canicola	0	10	10	10	10	1	0	10	n. a.
10	vaccine	sv Copenhageni	0	0	0	0	0	0	0	0^**^	n. a.
10	control	sg lcterohaemorrhagiae	0	10	10	8	4	1	0	10	n. a.
10	vaccine	sv lcterohaemorrhagiae	0	2	0	0	0	0	0	2^**^	2^**^
10	control	sg lcterohaemorrhagiae	0	9	6	2	0	0	0	9	17
10	vaccine	sv Bananal / Liangguan	0	0	0	0	0	0	0	0^**^	n. a.
9	control	sg Grippotyphosa	0	6	8	6	2	0	0	8	n. a.
8	vaccine	sv Grippotyphosa	0	0	0	0	0	0	0	0^**^	n. a.
10	control	sg Grippotyphosa	0	6	1	0	0	0	0	6	n. a.
10	vaccine	sv Bratislava	0	0	0	0	0	0	0	0^**^	n. a.
9	control	sg Austrais	0	6	4	0	0	0	0	6	n. a.
9	vaccine	sv Pomona	0	0	0	0	0	0	0	0^**^	n. a.
10	control	sg Pomona	0	10	10	10	9	2	0	10	n. a.
10	vaccine	sv vughia	0	0	0	0	0	0	0	0^**^	n. a.
10	control	sg Tarassovi	0	10	9	1	1	0	0	10	n. a.
10	vaccine	sv Tarassovi	0	2	0	0	0	0	0	2	2^*^
10	control	sg Tarassovi	0	6	2	1	1	0	0	6	10

^*^ *p* < 0.05. **p < 0.01

**Table 3:** Reisolation of Leptospira from urine and kidney. Pigs were vaccinated twice (4-week interval) and challenged with different *Leptospira* serovar (sv) and serogroup (sg) challenge strains, 6 weeks after first vaccination. Urine was sampled regularly and kidney samples were collected during necropsy 4w after challenge. A pig was considered shedding if at least once a positive urine isolation was found

			Reisolation of Leptospira from urine on post-challenge day						# pigs shedding	# kidney positive
n	Group	Challenge with	0	14	17	21	24	28		
9	vaccine	sv Canicola	0	0	0	0	0	0	0^**^	0^**^
10	control	sg Canicola	0	9	9	9	7	6	10	6
10	vaccine	sv Copenhageni	0	0	0	0	0	0	0	0
10	control	sg lcterohaemorrhagiae	0	0	0	0	1	1	1	0

**p < 0.01

**Table 4:** Leptospira strains used for vaccine / challenge

Species	Serogroup	Serovar	Strain	Originally isolated from
*Leptospira interrogans*	Canicola	Portland-vere^a^	Ca-I 2-000	human blood, 1964, Jamaica
*Leptospira interrogans*		Canicola^b^	Moulton	pig urine, 2004, Netherlands
*Leptospira interrogans*	lcterohaemorrhagiae	Copenhageni^a^	lc-02-001	rat, kidney, 1978, USA
*Leptospira interrogans*		Copenhageni^b^	CF1	dog, 1969, Puerto Rico
*Leptospira interrogans*		lcterohaemorrhagiae^b^	Verdun	human, 1917, France
*Leptospira kirschneri*	Grippotyphosa	Dadas^a^	Gr-01-005	kidney aborted piglet, 1983, USA
*Leptospira kirschneri*		Bananal/Lianguang^b^	11808	shrew, 1972, USA
*Leptospira kirschrneri*		Grippotyphosa^b^	142	horse eye, 1997 Germany
*Leptospira interrogans*	Australis	Bratislava^a^	As-05-073	pig placenta, 1989, USA
*Leptospira interrogans*		Bratislava^b^	X35lM-001	pig, 1990, USA
*Leptospira interrogans*	Pomona	Pomona^a^	Po-01-000	human blood, 1937, Australia
*Leptospira interrogans*		Pomona^b^	02-0162	not known
*Leptospira santarosai*	Tarassovi	Gatuni^a^	X345	human blood, 1938, Russia
*Leptospira weilii*		Vughia^b^	L100	pig kidney, 2001, China
*Leptospira borgpetersenii*		Tarassovi^b^	Perepelitsin	human blood, 1941, Russia

^a^vaccine strain

^b^challenge strain
